# Pre-treatment with Dazomet enhances the biocontrol efficacy of *purpureocillium lilacinum* to *Meloidogyne incognita*

**DOI:** 10.1186/s12866-023-02978-8

**Published:** 2023-08-31

**Authors:** Haizhen Nie, Binna Lv, Manhong Sun, Zengming Zhong, Shidong Li

**Affiliations:** 1 Institute of Plant Protection, Chinese Academy of Agricultural Sciences, Beijing, 100193 China; 2 Beijing Qigao Biological Technology Co. Ltd, Beijing, 100193 China

**Keywords:** Soil fumigation, *Purpureocillium lilacinum*, *Meloidogyne incognita*, Biocontrol, Synergistic effect

## Abstract

**Background:**

*Meloidogyne incognita* greatly restricts the production of protected vegetables in China. Application of biocontrol agent *Purpureocillium lilacinum* is an important practice to control the nematode; however, instability usually occurs especially in heavily infested field. This study aimed to illustrate the high efficiency of *P. lilacinum* agent with fumigant Dazomet in vitro.

**Results:**

*P. lilacinum* YES-2-14 showed strong parasitic and nematicidal activities to *M. incognita*. Pre-treatment with Dazomet significantly enhanced the biocontrol effects of the fungus. After fumigation with Dazomet at a dosage of 7.5 mg kg^− 1^ soil, parasitism of YES-2-14 on *M. incognita* eggs increased by more than 50%. Meanwhile, when *P. lilacinum* fermentation filtrate treated following Dazomet fumigation at 10 and 20 mg kg^− 1^ soil, the mortalities of second-stage juveniles (J_2_s) increased by 110.2% and 72.7%, respectively. Both Dazomet and *P. lilacinum* significantly reduced the penetration ability of J_2_s to tomato roots. When *P. lilacinum* filtrate used alone, the J_2_s penetrating into the young roots decreased by 48.8% at 4 dpi; while in the combined treatment, almost no J_2_ was detected within the roots at 4 dpi and the number of knots reduced by more than 99% at 45 dpi, indicating a synergistic effect of the biocontrol fungus and fumigant.

**Conclusions:**

Pre-treatment with Dazomet greatly increased the biocontrol efficacy of *P. lilacinum* to *M*. *incognita*. This research provides insight into the efficient management of plant parasitic nematodes and effective use of biocontrol agents.

## Background

Root-knot nematodes (*Meloidogyne* spp.) are destructive pathogens and cause huge losses in vegetable production due to their great varieties and wide distribution. In China, protected vegetable production has been developing rapidly and the area of facility planting has doubled in the past decade with the expansion of consumer demand [1]. However, monoculture and intensive input of chemical fertilizers have caused some serious problems including the degradation of soil ecosystem and the ensuing accumulation of various plant pathogens. It was estimated that approximately 50% of greenhouse-grown vegetables were infested by root-knot nematodes and the annual loss exceeded 400 million dollars [2].

Over the past few decades, methyl bromide was widely used and efficiently minimized the losses caused by root-knot nematodes [3–5]. Up to now, Dazomet, a fumigant with low toxicity and no damage to the stratospheric ozone layer, has been considered as an alternative to methyl bromide. The active ingredient of dazomet is its degradation product, methyl isothiocyanate, which can significantly reduce the populations of plant nematodes and pathogenic fungi and bacteria in soil [6–8]. However, repeated application of this fumigant may accelerate the degradation of the active ingredient methyl isothiocyanate (MITC), and therefore decrease the control efficiency to soil-borne diseases [9, 10]. Added to this, Dazomet cannot move through soil; therefore it must be mixed with soil and sufficient water thoroughly to be activated and avoid “hot spots” which may cause phytotoxicity due to high fumigant concentration [11–13].

Since the end of the 1990s, biocontrol agents have been playing increasingly important roles in the management of root-knot nematodes [14–17]. *Purpureocillium lilacinum* (syn. *Paecilomyces lilacinus*) is a promising nematophagous fungus which remarkably suppresses various plant nematodes by parasitizing eggs and females, producing cell wall degrading enzymes and nematicidal metabolites [18–22]. However, in seriously infested soil, application of the biocontrol agents sometimes was unsuccessful [23–25].

Strategies of combining different measures have been performed in the field to improve control efficiency to root-knot nematodes [26–28]. Martin [29] indicated that soil treatment with a low dosage of pesticides reduced the pressure of plant pathogens, then the newly introduced beneficial microorganisms could colonize easily and play a better role in the rhizosphere. There have been so far a few reports concerning the integrative management of root-knot nematodes by soil fumigants and microbial agents [30, 31]. Application of Dazomet followed by the antagonistic fungus *Pochonia chlamydosporia* and neem cake achieves the highest control efficiency to root-knot nematodes on rose in greenhouse, compared to the three practices used alone [32, 33]. In addition, soil fumigation with Dazomet at an early stage increases the population of beneficial bacteria introduced into soil afterwards [34]. The efficacies of Fosthiazate and Dazomet accompanied with *P. lilacinum* have also been confirmed in greenhouse experiments conducted over a 5-year period [35]. However, all these experiments were conducted in greenhouse or field without analysis of the primary cause. Tian et al. [36]. studied the synergistic mechanism of Dazomet and mycoparasite *Clonostachys rosea* conflicting against *Fusarium oxysporum* f. sp. *cucumerinum*, and demonstrated that severe damage occurred in the fungal pathogen when exposed to Dazomet-treated soil. Thus its endurance to the biocontrol fungus and infection potential to plant roots greatly weakened. However, the mechanism of integrated application of soil fumigants and biocontrol agents against plant nematodes is not yet clear.

Herein, we mainly focused on the evaluation of the synergistic effect of fumigant Dazomet and biocontrol agent *P. lilacinum* successively applied against *M*. *incognita* by in vitro tests and pot experiment. The findings provide an insight into the reduced application of chemical nematicides and effective use of biopesticides and sustainable management of plant parasitic nematodes in greenhouse vegetable production.

## Results

### Parasitism of YES-2-14 on ***M. incognita*** eggs

*P. lilacinum* YES-2-14 showed a strong parasitic capacity to *M*. *incognita* eggs and its parasitism increased significantly with the increasing of spore concentration (*P* < 0.01). When 6.5 × 10^5^ spores mL^− 1^ was used, the parasitic rate reached more than 80% and maintained the high level even at higher spore concentrations (Table 1).

**Table 1:** Parasitism of *P. lilacinus* YES-2-14 on *M. incognita* eggs

Concentration (spores mL^− 1^)	Parasitic rate (%)
0	0.0 ± 0.0 A
6.5 × 10^3^	35.1 ± 4.8 B
6.5 × 10^4^	56.5 ± 5.0 C
6.5 × 10^5^	84.0 ± 5.0 D
6.5 × 10^6^	83.8 ± 2.7 D
6.5 × 10^7^	84.6 ± 9.4 D

Data are means ± standard deviations of three replicates. Values followed by the same letters are not significantly different at *P* < 0.01 analyzed using Duncan’s new multiple range test.

### Effect of Dazomet on susceptibility of ***M. incognita*** eggs to ***P. lilacinum***

**Fig. 1:**
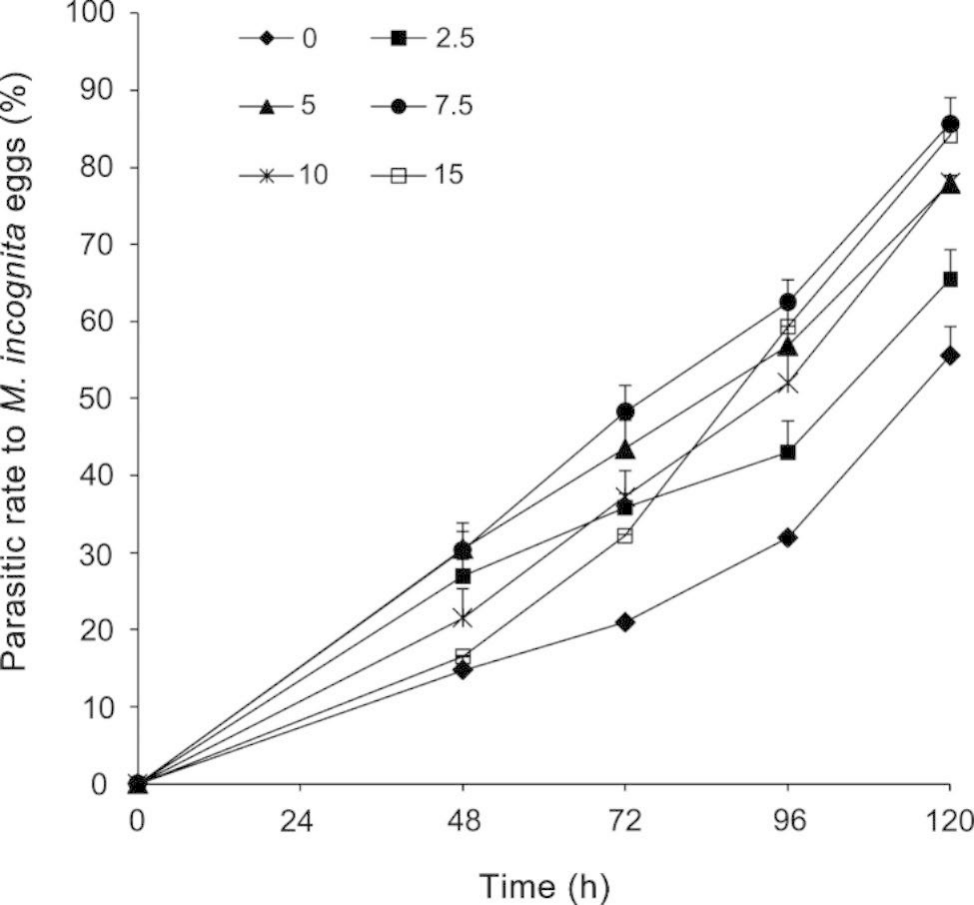
Parasitic rate of *P. lilacinum* YES-2-14 on pre-fumigated *M. incognita* eggs. A concentration of 6.5 × 10^4^ spore mL^− 1^ of *P. lilacinum* suspension was used. 0, 2.5, 5, 7.5, 10 and 15 respectively represent the fumigated nematode eggs with corresponding dosages of Dazomet for 3 days. The abscissa represents the incubation time of the pre-fumigated eggs with the spore suspension of *P. lilacinum*. Each point represents the mean value ± standard deviation of three independent replicates

To investigate the combined effect with the fumigant, *P. lilacinum* spore suspension with an initial concentration of 6.5 × 10^4^ spores mL^− 1^, which caused a moderate parasitic level of 56.5% on *M*. *incognita* eggs, was selected. As shown in Fig. 1, pre-treatment with Dazomet dramatically decreased the resistance of *M*. *incognita* eggs to *P. lilacinum*. When fungal treatment following fumigation with Dazomet at 5–15 mg kg^− 1^ soil, the percentages of eggs colonized by the nematophagous fungus reached 77.9 – 85.6% in 120 h, which were significantly higher than that of the un-fumigated control (*P* < 0.05). Furthermore, it was detected that the parasitic process initiated ahead of time considerably by pre-treating with Dazomet. *P. lilacinum* YES-2-14 needed 96 h to reach a high parasitic rate on the eggs pre-treated with Dazomet, which was approximately 24 h earlier than that on *M*. *incognita* eggs without fumigation.

During the whole parasitic process, the maximum synergistic effect of *P. lilacinum* and Dazomet, which was approximately twice the amount of the fungus used alone, was achieved when the concentration of the fumigant was 7.5 mg kg^− 1^ soil. For the eggs treated with higher dosages of Dazomet (10 and 15 mg kg^− 1^ soil), the parasitism was a little lower at early stage and rose rapidly and reached the highest level in 120 h (Fig. 1).

### Nematicidal activity of YES-2-14 fermentation filtrate and Dazomet on ***M. incognita*** J_2_s

*P. lilacinum* YES-2-14 demonstrated an intensive nematicidal activity to *M. incognita* J_2_s, which improved significantly with the concentration increase of fungal metabolites (*P* < 0.05). When equal volumes of J_2_ suspension and fungal filtrate were mixed, the mortality of J_2_s reached up to 84.2%, whilst the filtrate with 5-fold dilution resulted in a mortality of 25.5%. Even in the treatment with 10% fungal filtrate, the percentage of dead J_2_s was still 7.4%, which was significantly higher than that of the control (0.6%), in which culture broth without inoculation of *P. lilacinum* was used (Table 2). Given that a large number of live J_2_s were required for investigating their penetration ability into tomato roots, a 10-fold diluted fermentation filtrate was adopted for the following experiments.

**Table 2:** Nematicidal activity of *P. lilacinus* YES-2-14 fermentation filtrate on *M. incognita* J_2_s

Dilution ratio of filtrate	Mortality (%)	Corrective mortality (%)
CK	0.6 ± 1.0 a	0.0 ± 0.0 a
10	7.4 ± 0.6 b	6.9 ± 1.4 b
5	25.5 ± 4.9 c	25.1 ± 5.2 c
2	84.2 ± 4.6 d	84.1 ± 4.8 d

Data are means ± standard deviations of three replicates. CK represents the control of culture broth without inoculation of YES-2-14. Values followed by the same letters are not significantly different at *P* < 0.05 analyzed using Duncan’s new multiple range test.

Dazomet greatly suppressed the viability of *M. incognita* J_2_s, and the mortality of the juveniles rose significantly with the dosage increase of fumigant (*P* < 0.05, Table 3). Most *M. incognita* J_2_s were able to tolerate a certain dosage of no more than 10 mg kg^− 1^ soil, however, when 20 mg kg^− 1^ soil of Dazomet was applied, the mortality of J_2_s reached over 50%. In addition, Dazomet fumigation significantly decreased the tolerance of the juveniles to YES-2-14 filtrate (*P* < 0.05, Table 3). When the combined treatments of 10 and 20 mg kg^− 1^ soil of Dazomet followed by 10-fold diluted YES-2-14 filtrate were used, the mortalities of J_2_s increased by 110.2% and 72.7%, respectively, compared to the theoretical mortalities (Table 3). In order to reduce the usage of Dazomet and get a number of live J_2_s for the subsequent pathogenicity assay, a dosage of 10 mg kg^− 1^ soil was selected.

**Table 3:** Nematicidal activity of Dazomet and its effect on the endurance of *M. incognita* J_2_s to the fermentation filtrate of *P. lilacinum* YES-2-14

Dosage of Dazomet (mg kg^− 1^ soil)	J_2_ mortality in Dazomet treatment (%)	J_2_ mortality in combined treatment (%)^#^	*P*	*M* (%)^†^
0	0.7 ± 0.1 a	7.4 ± 1.4 a	0.0156	7.4
2.5	2.2 ± 0.8 ab	8.5 ± 1.0 a	0.0012	8.8
5	3.1 ± 2.4 b	10.0 ± 0.5 a	0.0082	9.6
10	5.4 ± 1.7 c	24.8 ± 3.1 b	0.0007	11.8
20	52.1 ± 1.5 d	95.0 ± 1.3 c	< 0.0001	55.0

Data are means ± standard deviations of three independent replicates. Values within a column followed by the same letter are not significantly different at *P* < 0.05 level according to Duncan’s multiple range test.

# Values in this column represent J_2_ mortality caused by the combined treatment of Dazomet fumigation followed by the 10-fold diluted fermentation filtrate of *P. lilacinum* YES-2-14.

*P* represents statistical difference between J_2_ mortality post fumigation and J_2_ mortality post fumigation and fungal filtrate using the *t* test.

^†^*MT* represents the theoretical mortality of J_2_s treated with Dazomet and *P. lilacinum* successively. *MT* (%) = 1 - (1-*mr*) × (1- *mo*), in which *mr* and *mo* represent the revised mortality of J_2_s in the treatments of Dazomet fumigation and fungal filtrate respectively.

### **Effects of Dazomet and*P. lilacinum*on pathogenicity of J**_**2**_**s to tomato**

**Fig. 2:**
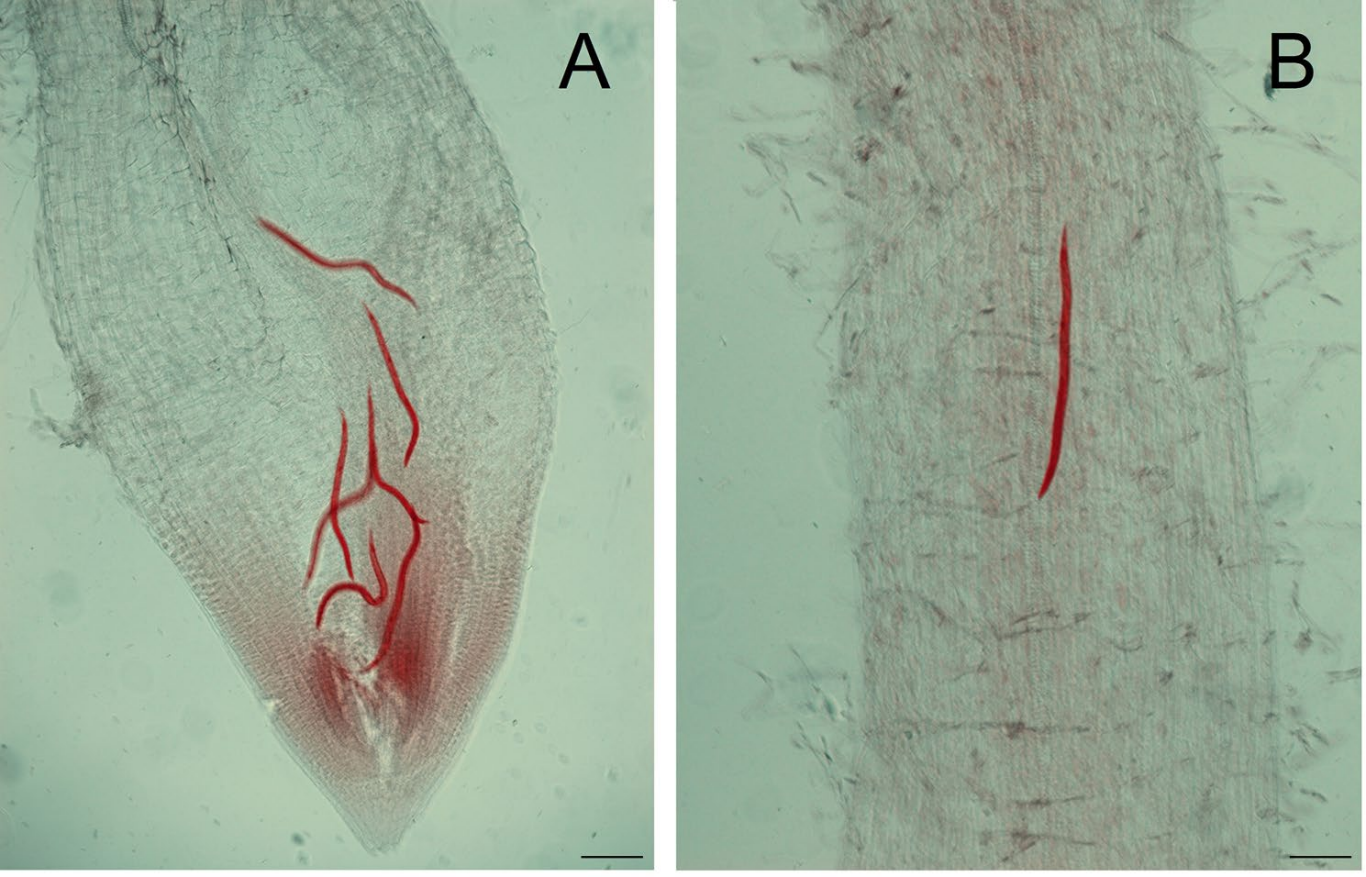
*M. incognita* J_2_s penetrated into tomato young roots at 4 dpi. The sample was stained using acid fuchsin and bleached using glycerin. (A) J_2_s in the elongation zone of tomato root tip; (B) J_2_s in the maturation zone of tomato root tip. Scale bars = 100 μm

*M. incognita* J_2_s preferred to penetrate from the tender tissues of tomato root tip and establish its permanent feeding sites within the inner cells of the host (Fig. 2).

**Fig. 3:**
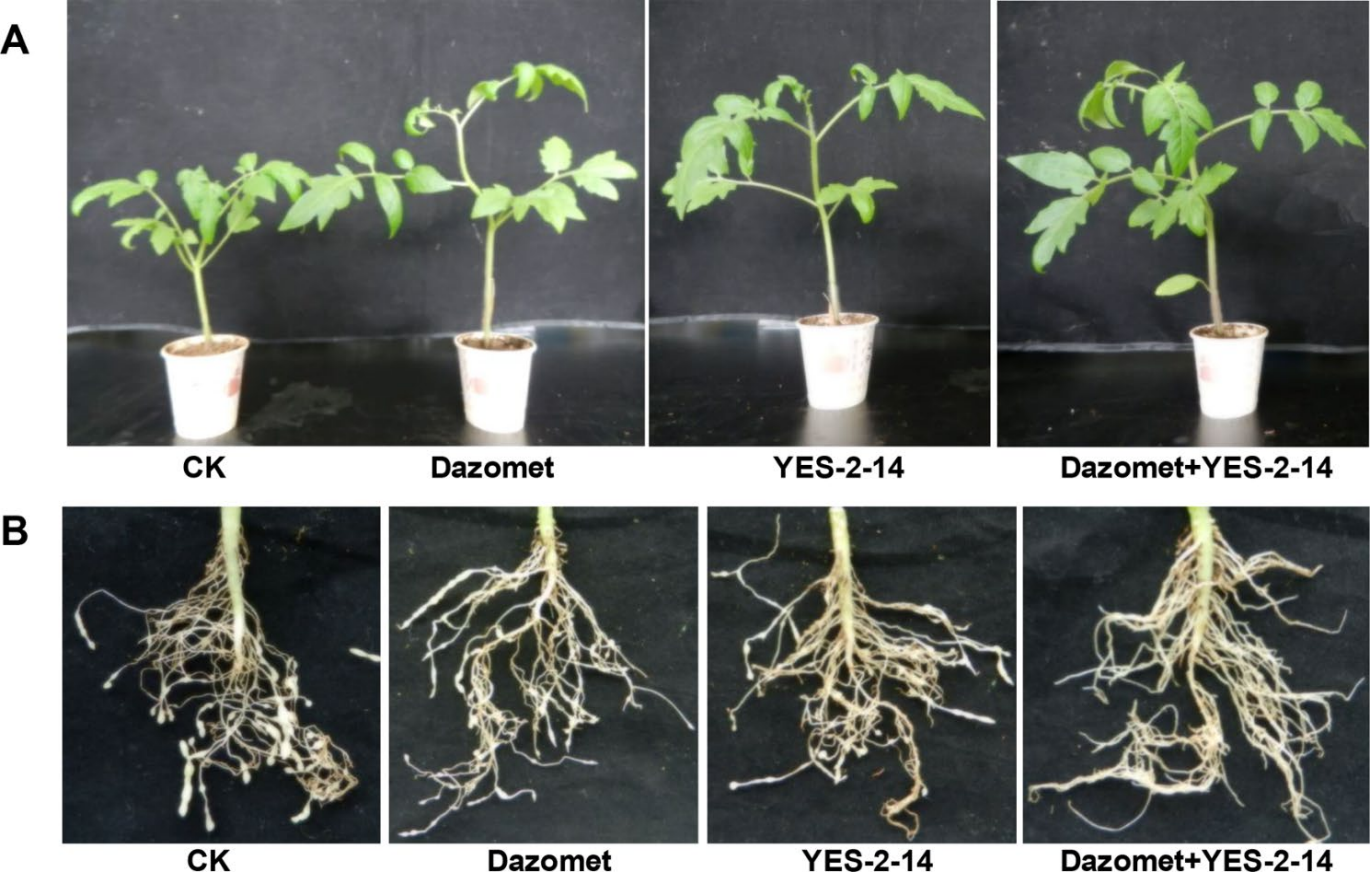
Effects of *M. incognita* J_2_s treated with Dazomet and *P. lilacinum* YES-2-14 on tomato seedlings and knot formation on the roots at 45 dpi. (A) Tomato seedlings infested by treated J_2_s; (B) Knots on tomato roots infested by treated J_2_s. CK, Dazomet, YES-2-14 and Dazomet + YES-2-14 respectively represent the tomato seedlings inoculated with *M. incognita* J_2_s treated with these four different treatments

The vigor of tomato seedlings that were inoculated with J_2_s treated by Dazomet and *P. lilacinum* filtrate was significantly stronger than that of the untreated control. Furthermore, the penetration of the juveniles and the formation of knots on tomato roots were notably suppressed under the co-treatment of both agents (Fig. 3). When the fermentation filtrate of YES-2-14 was used at 10-fold dilution, the number of J_2_s penetrating into the young roots and the knots formed on roots reduced by 48.8% and 23.9%, respectively, compared to the control. However, if the fungal treatment following Dazomet fumigation at 10 mg kg^− 1^ soil was used, no J_2_s were detected in tomato roots in the early stage and the number of knots per root reduced by more than 99%, indicating that the combined use of Dazomet and biocontrol agent *P. lilacinum* was a highly efficient practice to inhibit the infestation of *M. incognita* and the formation of knots on plant roots (Table 4).

**Table 4:** Effects of Dazomet and *P. lilacinum* YES-2-14 on the penetrating activity and pathogenicity of *M. incognita* J_2_s on tomato roots

Treatment	No. J_2_s root^− 1^ (4 dpi)	No. knots root^− 1^ (45 dpi)
CK	160 ± 11 A	377 ± 27 A
Dazomet	16 ± 5 C	106 ± 35 C
YES-2-14	82 ± 5 B	287 ± 19 B
Dazomet + YES-2-14	0 ± 0 D	1 ± 0 D

Data are means ± standard deviations of three independent replicates. Different letters in the same column are significantly different at *P* < 0.01 according to Duncan’s multiple range test. CK, Dazomet, YES-2-14 and Dazomet + YES-2-14 represent the tomato seedlings were inoculated with *M. incognita* J_2_s treated with the four different treatments. dpi, days post inoculation.

## Discussion

Biological control is playing an increasing role in the management of root-knot nematodes in vegetable production. However, unstable efficacy sometimes occurs when nematode density is very high [37, 38]. After long periods of planting, especially monoculture of high-value vegetables in protected greenhouse, plant nematodes and other harmful microorganisms accumulate massively in soil, leading to difficulties for the introduced beneficial organisms to survive and combat with the indigenous pathogens [39, 40]. Dazomet fumigation can greatly eliminate various plant pathogens and thus reduce their infection potential in a short period of time. Under this situation, the newly introduced microorganisms are more likely to germinate, colonize, and establish a large population in the rhizosphere rapidly to attack the residual pathogens and suppress their proliferation and invasion [29, 41].

The efficiency of biocontrol fungi applied together with chemical fumigants on soilborne diseases has been proven in some field experiments [4]. However, the root causes of the highly efficiency of the combined measures have seldom been touched. In this study, fumigant Dazomet and biocontrol agent *P. lilacinum* YES-2-14 were applied successively on the eggs and J_2_s of *M. incognita in vitro* to illuminate the mechanism of the combined practice. The results revealed that pre-treatment with Dazomet significantly reduced the resistance of *M. incognita* to *P. lilacinum* and infestation to tomato seedlings. Treated with the fumigant, nematode eggs and J_2_s were stifled to death or injured and paralyzed, bringing a weakening in their immune defense and a significantly decreased tolerance to extraneous pressure of *P. lilacinum*. Although a same amount of live J_2_s were picked to inoculate tomato roots, the co-treated ones appeared to show a much lower invasive ability and pathogenicity on tomato plants compared to the singly treated and healthy inocula. The obtained results were consistent with the study showing the synergistic mechanism of Dazomet and biocontrol agent *Clonostachys rosea* against cucumber wilt caused by *Fusarium oxysporum* f. sp. *cucumerinum* [36]. Analyzed with the Bliss independent model [42], which is often used to analyze the correlation among different biocontrol agents or different control measures in plant disease management [43, 44], an obvious synergistic effect between Dazomet and *P. lilacinum* was achieved in the present study.

*P. lilacinum* is a facultative fungus widely distributed in soil. When in contact with nematode eggs or females, fungal spores germinate quickly, penetrate the cuticle or egg shell with its invasive hyphae, and thereby begin its parasitic lifestyle as other nematode-parasitic and nematode-trapping fungi [45, 46]. Thus to a certain extent, the viability of nematode eggs might influence the nutrition mode of the associated fungus, which may experience the adaption from parasitism to saprophytism [47]. After fumigation with Dazomet at 7.5 mg kg^− 1^ soil, the numbers of eggs infected by YES-2-14 were the highest throughout the 120 h, indicating a strong parasitic ability of *P. lilacinum* to the suppressed nematode eggs. However, for the eggs exposed to higher dosages of fumigant, parasitism was weak at earlier stages, but increased and finally reached a level equivalent to that obtained with Dazomet at 7.5 mg kg^− 1^ soil. It is speculated that when fumigated with high dosages of Dazomet, a number of *M. incognita* eggs might be dead, on which the nematophagous fungus feeds itself and switches into a necrotrophic phase [48].

The second-stage juvenile is the only infectious form in the whole life cycle of *M. incognita*. Once penetrating into plant roots, they establish permanent feeding sites within the central vascular cylinder [49] and cause serious systemic disease. Therefore, management practices conducted before the initial infestation are crucial for the control of root-knot nematodes. Dazomet acts as a biocide by releasing methyl isothiocyanate when applied in soil with the addition of water, while the fungus *P. lilacinum* is capable of parasitizing nematode eggs and females and secreting nematicidal metabolites such as lower fatty acids and leucinostatin [50–52]. In this study, both Dazomet and *P. lilacinum* demonstrated strong suppressive effects on *M. incognita*. When used in successive combination, the survival of J_2_s and their infectivity significantly decreased compared to the two agents used alone. Moreover, few J_2_s and knots were detected in and on tomato roots in the treatment of *P. lilacinum* following Dazomet fumigation at lower concentration. Hereafter, an integrated management approach of reduced dose of chemical fumigants integrated with biocontrol agents could be applied widely in the field to suppress plant nematodes and achieve sustainable vegetable production.

## Conclusion

We have found, for the first time, the synergistic effect of fumigant Dazomet and biocontrol fungus *P. lilacinum* against *M. incognita*. Pre-treatment with Dazomet significantly reduced the endurance of *M. incognita* eggs and J_2_s to *P. lilacinum* YES-2-14. The two agents used in successive combination significantly suppressed the penetration and pathogenicity of J_2_s to tomato roots and achieved a much higher control efficiency on root-knot nematodes. This study provides an insight into the reduced use of chemical pesticides and effective use of biopesticides to manage plant nematodes efficiently.

## Methods

### Isolate

*P. lilacinum* YES-2-14 was originally isolated from egg masses associated with tobacco roots in Ershan County, Yunnan Province, China. This strain is maintained in the Biocontrol of Soil-borne Diseases Lab in the Institute of Plant Protection, Chinese Academy of Agricultural Sciences (CAAS) and the Institute of Microbiology, Chinese Academy of Sciences (CAS).

### Soil fumigant

Dazomet powder (98%) was provided by the Applied Microbiology Lab at the Institute of Plant Protection, Shanghai Academy of Agricultural Sciences.

### Plant cultivar

The tomato cultivar Jiabao F1 was provided by Beijing Jingyu Kingwinner Agriculture S&T Co., Ltd. This cultivar is susceptible to root-knot nematodes.

### **Preparation of spore suspension and fermentation filtrate of*P. lilacinum***

Culture blocks (ca. 6 mm) of YES-2-14 grown on potato dextrose agar (PDA) plate for 7 days were cut with a sterile puncher and put into a 50 mL centrifuge tube with 10 mL sterile water. Then the tube was shaken vigorously on a vortex mixer for 10–15 s and the liquid was passed through a sterile sieve (25 μm) to remove mycelial fragments. The spore suspension was collected in a sterile plastic tube and the concentration was determined under a microscope (BX41, Olympus, Tokyo, Japan) with a hemocytometer.

Four agar blocks (ca. 6 mm) of YES-2-14 were inoculated into 100 mL liquid medium (25.0 g sucrose, 10.0 g yeast extract powder, 1.0 g K2HPO4, 0.5 g NaCl, 0.5 g MgSO4·7H2O and 50 mg ZnSO4·7H2O per liter) in a 500 mL flask and incubated at 26ºC on a fermentation shaker at a speed of 180 rpm. After 72 h, the acquired fermentation liquor was centrifuged at 8000 rpm for 10 min, and the supernatant was recovered and passed through a sterile 0.25 μm Millipore filter to prepare the fermentation filtrate.

### **Preparation of egg and juvenile suspension of*M***. ***incognita***

*M*. *incognita* was propagated on tomato plants in greenhouse. Heavily infested roots were collected and the nematode eggs were harvested according to the hypochlorite method [53] with a few modifications. Briefly, the infested roots, with knots and egg masses, were washed with tap water, cut into 2–3 cm fragments and disinfected with 0.5% NaClO for 2 min. The suspension was poured successively through a series of sieves with 250-, 75- and 25-µm pore, and rinsed with sterile distilled water repeatedly. The eggs on the 25-µm-pore sieve were transferred into a sterile centrifuge tube, and the concentration of the egg suspension was determined by triplicate counts under an inverted microscope (CK2, Olympus, Tokyo, Japan) and adjusted to 2000 and 300 eggs per milliliter respectively with sterile distilled water for subsequent bioassays.

*M*. *incognita* J_2_s were obtained basing on the method of Siddiqui et al. [54]. Freshly obtained eggs were placed on a 25-µm-pore sterile sieve with a sterile tray below. Sterile water was added to the tray to just touch the lower portion of the sieve. The assembly was placed in an incubator at 25ºC for 2–3 days and the hatched juveniles were collected from the tray at an interval of 24 h. The concentration of the newly obtained J_2_ suspension was measured under an inverted microscope.

### Soil fumigation

The field practice of the fumigant was simulated in the lab to determine the effects of Dazomet on eggs and juveniles of root-knot nematode in vitro. The soil was collected from an experimental vegetable yard of the Institute of Plant Protection, CAAS, in Langfang, Hebei Province, China, air-dried and passed through a 1-mm-pore sieve. Then 1000 g soil was mixed thoroughly with a certain dosage of Dazomet powder and poured into a 2-liter desiccator. A certain amount of sterile water was added to reach 30% of water content to ensure the optimal degradation and dissipation of Dazomet. The container was sealed immediately with Vaseline and placed in an incubator at 26 °C.

### **Parasitism of*P. lilacinum*on*M. incognita*eggs**

Five concentrations of *P. lilacinum*, 6.5 × 10^3^, 6.5 × 10^4^, 6.5 × 10^5^, 6.5 × 10^6^ and 6.5 × 10^7^ spores mL^− 1^, were used to evaluate its parasitism to *M*. *incognita*. 1 mL spore suspension and equal volume of egg suspension (ca. 300 eggs) were mixed in a 24-well tissue culture plate [55]. After incubating for 5 days at 26 °C, the infected and uninfected eggs were counted under an inverted microscope using a counting dish. The eggs treated with sterile water were taken as the control, and four replicates were conducted. The bioassay was repeated three times using different sets of fungal suspension and egg suspension.

### **Resistance of fumigated eggs to*P. lilacinum***

Aliquots of newly prepared egg suspension (ca. 2000 eggs) were dropped into sterile packets (3 cm × 2 cm) made of 25-µm-pore nylon cloth. The packets were submerged into soils mixed with Dazomet at 0, 2.5, 5.0, 7.5, 10.0, and 15.0 mg kg^− 1^ soil, respectively, in the desiccators. After fumigating at room temperature for 3 days, the small packets were taken out and washed with sterile distilled water to get rid of the soil. The eggs were recovered from each packet and the concentrations were adjusted to 300 eggs mL^− 1^.

*P. lilacinum* spore suspension with a concentration causing moderate parasitism on *M*. *incognita* eggs was adopted to investigate its effect on fumigated eggs. The retrieved eggs were mixed with the fungal suspension thoroughly in the holes of 24-well plates. The parasitized and non-parasitized eggs were counted under an inverted microscope at 100 × magnification from 48 h up to 120 h. Three replicates for each treatment were used and the bioassay was conducted three times using different sets of *M*. *incognita* eggs and fungal suspension.

### Nematicidal activity of Dazomet in vitro

Two thousand newly hatched juveniles were enclosed into 15-µm-pore nylon packets (3 cm × 2 cm) and fumigated with Dazomet at five doses, 0, 2.5, 5.0, 10.0, and 20.0 mg kg^− 1^ soil. The J_2_s were recovered after fumigation at room temperature for 3 days and their viability was examined immediately according to the staining method [56] with some modifications: 1–2 drops of 0.5% Safranine O solution were added to 2 mL J_2_ suspension and mixed thoroughly at room temperature for 30 min. The juveniles dyed red were regarded to be dead, while the uncolored alive. Three replicates were conducted and the experiment was run three times.

### **Nematicidal activity of*P. lilacinum*fermentation filtrate**

The fermentation filtrate of YES-2-14 was serially diluted 0, 2.5 and 5 times and aliquots of 1 mL were respectively mixed with equal volume of juvenile suspension (ca. 300 J_2_s) in 24-well plates. The plates were placed at 26 °C for 3 days and the dead and live juveniles were distinguished and counted as described above. The liquid broth without inoculation of *P. lilacinum* acted as the control, and three replicates for each treatment were conducted. The bioassay was repeated three times with new sets of fungal filtrates and J_2_ suspension.

### **Endurance of fumigated J**_**2**_**s to*P. lilacinum***

An appropriate dilution ratio of the fungal filtrate was adopted to investigate its nematicidal activity on pre-fumigated juveniles. The fresh J_2_s were fumigated with different dosages of Dazomet, 0, 2.5, 5.0, 10.0, and 20.0 mg kg^− 1^ soil, for 3 days at room temperature. Subsequently, the recovered J_2_s were mixed with fungal filtrate in a 24-well plate and incubated at 26 °C for 3 days. The viability of the doubly treated J_2_s was determined and the theoretical mortality (*MT*) was calculated using the equation: *MT* (%) = 1 - (1-*mr*) × (1- *mo*), in which *mr* and *mo* represent the revised mortality of J_2_s after Dazomet fumigation and fungal filtrate treatment. Three independent replicates were conducted for each treatment and the bioassay was run three times using different sets of nematicidal agents and J_2_s.

### **Effects of Dazomet and*P. lilacinum*on pathogenicity of*M***. ***incognita*J**_**2**_**s**

The suppressive effects of Dazomet (10 mg kg^− 1^ soil) and *P. lilacinum* (1:10 diluted filtrate) used individually and successively on the penetration of J_2_s to tomato roots were evaluated using pot experiment in greenhouse. Firstly, pre-treated J_2_s were prepared and incubated with YES-2-14 filtrate as mentioned above. Four treatments were set: (1) CK, treatment in soil without Dazomet followed by incubation in liquid medium; (2) Dazomet, Dazomet fumigation followed by incubation in liquid medium; (3) YES-2-14, treatment in soil without Dazomet followed by incubation in YES-2-14 filtrate; (4) Dazomet + YES-2-14, Dazomet fumigation followed by incubation in fungal filtrate. Subsequently, J_2_s from each treatment were recovered and the live were counted for the following inoculation of tomato roots.

The tomato cultivar Jiabao F1 was planted in seed trays at 20ºC–28ºC. After growing for 3 weeks, the tomato seedlings were transferred into 5-cm deep holes in autoclaved sandy loam in pots (φ 9 cm). Simultaneously, 500 live J_2_s newly recovered from each treatment were inoculated in the rooting-zone. There was one seedling in each pot, 18 pots per treatment. The pots were kept in the greenhouse at 28ºC in the day and 20ºC at night, and irrigated daily. The test was conducted in a randomized complete block design with three independent biological replicates.

Half of the seedlings were uprooted at 4 dpi (days post inoculation) and rinsed with tap water to get rid of the soil. Then the tender roots were cut off, disinfected with 1% NaClO for 4 min, rinsed with sterile distilled water repeatedly, and sopped up with sterile filter paper. The root tissues were stained with acid fuchsin and bleached with acid glycerin according to the method of Bybd et al. [57] except that the samples were heated in a microwave oven at a low power for 2 min instead of boiling during the staining and decoloration processes. The tomato roots were examined under a microscope (100 × magnification) and the J_2_s penetrating into the plant roots were counted. At 45 dpi, the other half of the seedlings were uprooted and the knots formed on the roots were counted. The bioassay was conducted three times, each time with three biological replicates.

### Statistical analysis

Data from all the bioassays were processed with SAS (Statistics Analysis System) version 9.2 (SAS Institute Inc, Kerry. State of North Carolina, USA). Analysis of variance (ANOVA) and *t* test were used to determine the difference between the means from each treatment.

## Data Availability

All data and materials used in this study are available from the corresponding author upon request.
